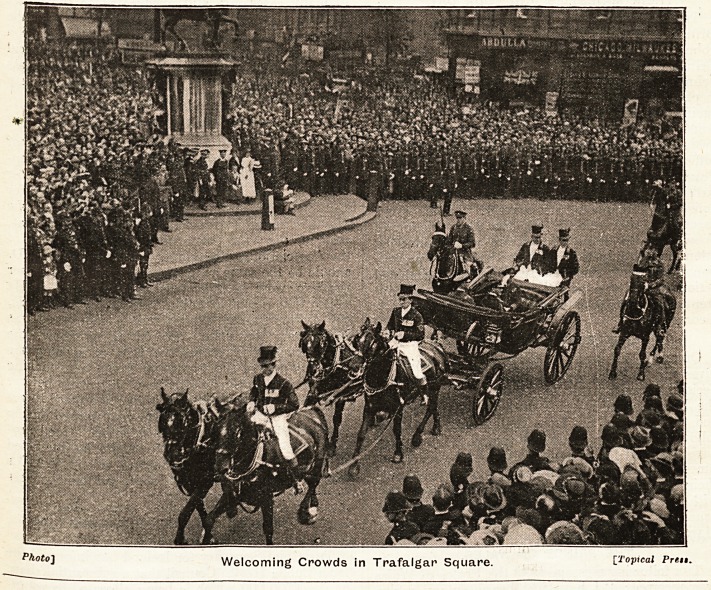# A Memorable Home-Coming

**Published:** 1920-10-16

**Authors:** 


					October 16, 1920. THE HOSPITAL. 45
A Memorable Home-Coming.

				

## Figures and Tables

**Figure f1:**